# A Modified Particle Swarm Optimization Technique for Finding Optimal Designs for Mixture Models

**DOI:** 10.1371/journal.pone.0124720

**Published:** 2015-06-19

**Authors:** Weng Kee Wong, Ray-Bing Chen, Chien-Chih Huang, Weichung Wang

**Affiliations:** 1 Department of Biostatistics, University of California, Los Angeles, USA; 2 Department of Statistics, National Cheng Kung University, Taiwan; 3 Department of Mathematics, National Taiwan University, Taipei, Taiwan; 4 Institute of Applied Mathematical Sciences, National Taiwan University, Taiwan; University of Minnesota, UNITED STATES

## Abstract

Particle Swarm Optimization (PSO) is a meta-heuristic algorithm that has been shown to be successful in solving a wide variety of real and complicated optimization problems in engineering and computer science. This paper introduces a projection based PSO technique, named ProjPSO, to efficiently find different types of optimal designs, or nearly optimal designs, for mixture models with and without constraints on the components, and also for related models, like the log contrast models. We also compare the modified PSO performance with Fedorov's algorithm, a popular algorithm used to generate optimal designs, Cocktail algorithm, and the recent algorithm proposed by [1].

## Introduction

Mixture experiments are widely used in food processing, chemical, manufacturing, agricultural, cosmetics and pharmaceutical industries. For example, [2, 3] and [4] analyzed data from optimal designs for different mixture models in the pharmaceutical industry. Interest in mixture models is growing because such models are increasingly used in various fields and also optimal designs are becoming more available via computer codes, software packages and interactive online websites. [5] provides an excellent introduction and broad coverage in mixture experiments. A review of mixture models and their optimal designs can be found in [6] and research work in mixture experiments over the last 50 years is reviewed by Pipel in Chapter 12 of an edited monograph [7].

The aim of this paper is to introduce a popular optimization technique already widely used in engineering and computer science research and modify it to find various optimal designs for different types of mixture models. Particle Swarm Optimization (PSO) techniques have been around for more than ten years, but interestingly haven’t made much impact in statistical applications to date. Our experience reinforces the widely held findings that PSO is a very simple and powerful optimization tool. It requires no assumption of the objective function and has only a few easy-to-use tuning parameters. PSO is intriguing because even though the theory is not fully developed, its repeated successes and increasing widespread applications in various disciplines have even resulted in at least 3 journals that mainly track PSO development and applications in various fields.

Section 2 provides statistical setup and briefly reviews general theory for optimal experimental designs before discussing mixture models and their optimal designs. In Section 3, we propose a PSO-based method for finding a variety of optimal designs for different types of mixture experiments. In Section 4 we show that this procedure is efficient for finding various types of optimal or nearly optimal designs for different types of mixture models, including several new models for which optimal designs are not known analytically. Section 5 compares PSO performance with the popular Fedorov-exchange algorithms for finding optimal designs and we conclude in Section 6 with a summary of the advantages of the proposed PSO method over current methods for generating optimal designs.

## Background

Our interest is in the general linear model given by

$$\begin{array}{c} y=\beta^{\prime }f\left(x\right)+\epsilon ,\;\;\;\;x\in \Omega . \end{array}$$ (1)

Here *y* is the response variable, ***β*** is a *d*×1-vector of unknown coefficients, *f*(**x**) is a given *d*×1 vector of linearly independent regression functions defined on a user-defined compact design space Ω. The error *ϵ* has zero mean and constant variance and we assume all errors are normally and independently distributed. An approximate design *ξ* is defined by its design points (**x**_*i*_’s) and the proportions (*p*_*i*_’s) of observations to be taken at these points. Once the sample size *n* is fixed, either by cost or time considerations, and an optimality criterion is given, the problem is to determine the number (*k*) of points required, along with the values of **x**_*i*_ and *p*_*i*_, *i* = 1, …, *k* that optimize the criterion. The implemented design takes roughly *np*_*i*_ observations at **x**_*i*_, *i* = 1 …, *k* from Ω subject to *np*_1_ + … + *np*_*k*_ = *n* and each *np*_*i*_ is a positive integer.

Following convention, the worth of a design *ξ* is measured by its total Fisher information matrix, which is obtained by taking the negative of the expectation of the second derivative of the logarithm of the likelihood function with respect to ***β***. For Eq (1), this matrix is **M**(*ξ*) = *E*_**ξ**_(*f*(**x**)*f*(**x**)′), which is inversely proportional to the variance-covariance matrix of the estimated parameters *β*.

The design criterion is formulated as a convex function of the information matrix. For example, *D*-optimality for estimating model parameters seeks to minimize the generalized variance using the convex functional Φ(*ξ*) = −*ln* |*M*(*ξ*)|. Another popular and useful criterion is *L*-optimality defined by Φ(*ξ*) = *tr* *L* *M*(*ξ*)^−1^ and *L* is a user-selected matrix; if the goal is to minimize the average of the variances of the estimated parameters, we set *L* = *I*, the identity matrix whereupon *L*-optimality reduces to *A*-optimality. Alternatively, if the goal is to estimate some average of the response over a user-selected region *R*, one chooses *L* = ∫_*R*_ *f*(**x**)*f*′(**x**)*μ*(*d* **x**) and *μ* is a a selected weighting measure over for *R*. This corresponds to estimating the response surface over *R* with weights specified by the measure *μ* with more important parts of *R* receiving a larger weight. If there is equal interest over the region *R*, one chooses *μ* to be the uniform measure on *R*. We obtain *I*-optimality when *μ* is the uniform measure and *R* = Ω.

Given a statistical model and a convex design criterion, [8] gave us a tool to verify whether an approximate design is optimal among all designs on a known compact design space Ω. For example, for Eq (1),

- a design *ξ* is *D*-optimal if *f*(**x**)′ *M* ^−1^(*ξ*)*f*(**x**) ≤ *d* for all **x** ∈ Ω, and
- a design *ξ* is *L*-optimal if *f*(**x**)′ *M* ^−1^(*ξ*) *L* *M* ^−1^(*ξ*)*f*(**x**) ≤ tr*L* *M* ^−1^(*ξ*) for all **x** ∈ Ω.

These are frequently referred to as equivalence theorems or more informally as checking conditions. They are derived from considerations of the Frechet derivatives of the convex functionals at the optimum; the function on the left hand side of each of the above inequality is the directional derivative of the convex design criterion evaluated at the optimum. When the regression model has one or two independent variables, the equivalence theorem can be easily applied to check the optimality of any design graphically. For instance, to check whether a design *ξ* is *D*-optimal, we plot the function on the left hand side of the inequality (a) over the design space and determine whether the inequality is satisfied. If it is, the design *ξ* is *D*-optimal; otherwise it is not.

The worth of a design *ξ* is measured by its efficiency relative to the optimum. Typically, this measure is the ratio of the criterion values of the two designs or some function of this ratio thereof. For instance, the *L*-efficiency of *ξ* is *tr* *L* *M*(*ξ*_*L*_)^−1^/*tr* *L* *M*(*ξ*)^−1^, where *ξ*_*L*_ is the *L*-optimal design. If this ratio is 0.5, then *ξ* has to be replicated twice to perform as well as the *L*-optimal design *ξ*_*L*_. To maintain this interpretation, *D*-efficiency of a design *ξ* is defined by |*M*(*ξ*)*M* ^−1^(*ξ*_*D*_)|^1/*d*^ where *ξ*_*D*_ is the *D*-optimal design and *d* is the dimension of the regression function *f*(*x*). When the equivalence theorem shows that a design is not optimal, we can also assess its proximity to the optimal (without knowing the optimum) using an efficiency lower bound derived from the equivalence theorem and an examination of the above plot [9]. This is helpful when an algorithm takes too long to converge to the optimum or is terminated prematurely when it reaches the maximum pre-specified number of iterations and we wish to ascertain the efficiency of the generated design. We illustrate such situations in subsection 4.5 where we consider design problems for mixture experiment with constraints on the components.

### Mixture Models

It appears much of the recent design work for mixture models focuses on finding designs robust to model mis-specification. For example, [10] constructed *A*-optimal designs for mixture experiments that are robust to the linear and quadratic models proposed by [11]. In addition, [12] and, [13] found *D*- and *A*-optimal designs for linear log contrast and quadratic log contrast models for experiments with mixtures, respectively. [14] advocated a trace criterion to estimate the best proportions for the ingredients or components and [15] explored a minimax criterion to estimate the response surface in a mixture experiment, including using a deficiency criterion to measure the goodness of a mixture experiment. In both papers, the model was a quadratic polynomial in several factors over the simplex region.

We assume our mixture experiments have *q* factors *x*_1_, *x*_2_, …, *x*_*q*_ defined on the regular *q* simplex $S^{q-1}=\{{\text{x}}^{\prime }=(x_{1},x_{2},\ldots ,x_{q})\in {\left[0,1\right]}^{q}:\sum_{i=1}^{q}x_{i}=1\}$. Some of the most common mixture models used in practice are Scheffé’s polynomials of order *n*. If *ϵ* denotes random error, the simplest is an additive polynomial mixture model when *n* = 1 and *f*(**x**)′ = (*x*_1_, *x*_2_, …, *x*_*q*_) given by

$$\begin{array}{c} y=\beta^{\prime }x+\epsilon =\sum_{i=1}^{q}\beta_{i}x_{i}+\epsilon . \end{array}$$ (2)

When *n* = 2 and *f*(**x**)′ = (*x*_1_, *x*_2_, …, *x*_*q*_, *x*_1_ *x*_2_, *x*_1_ *x*_3_, …, *x*_*q*−1_ *x*_*q*_), the second degree Scheffé’s polynomial mixture model is

$$\begin{array}{c} y=\sum_{i=1}^{q}\beta_{i}x_{i}+\sum_{1\leq i<j\leq q}\beta_{ij}x_{i}x_{j}+\epsilon . \end{array}$$ (3)

This is an example of a Scheffé quadratic canonical polynomial models widely used in blending experiments in engineering, agriculture, biological and the medical sciences. In the notation of Eq (1), we have *d* = *q* for Eq (2) and *d* = *q* + *q*(*q* + 1)/2 for Eq (3). More generally, the Scheffé polynomial of order *n* for a *q*-component mixture model is

$$\begin{array}{c} y=\sum_{i=1}^{q}\theta_{i}x_{i}+\sum_{1\leq i<j\leq q}\phi_{ij}x_{i}x_{j}+\cdots +\sum_{1\leq i_{1}<\cdots <i_{n}\leq q}\phi_{i_{1}\cdots i_{n}}x_{i_{1}}\cdots x_{i_{n}}+\epsilon . \end{array}$$ (4)

[16, 17] proposed a class of flexible models for studying mixture experiments with additive effects when the mean response also depends linearly on the total amount used in the experiment. A requirement is that all components in the regression function are homogenous of degree 1. Becker’s models include

$$\begin{array}{c} y=\sum_{i=1}^{q}\theta_{i}x_{i}+\sum_{i<j}\phi_{ij}\min\left(x_{i},x_{j}\right)+\cdots +\phi_{1,2,\cdots ,q}\min\left(x_{1},\cdots ,x_{q}\right)+\epsilon , \end{array}$$ (5)

$$\begin{array}{c} y=\sum_{i=1}^{q}\theta_{i}x_{i}+\sum_{1\leq i<j\leq q}\frac{\phi_{ij}x_{i}x_{j}}{x_{i}+x_{j}}+\cdots +\frac{\phi_{1,2,\cdots ,q}x_{1}x_{2}\cdots ,x_{q}}{{\left(x_{1}+\cdots +x_{q}\right)}^{q-1}}+\epsilon , \end{array}$$ (6)

$$\begin{array}{c} y=\sum_{i=1}^{q}\theta_{i}x_{i}+\sum_{1\leq i<j\leq q}\phi_{ij}{\left(x_{i}x_{j}\right)}^{1/2}+\cdots +\phi_{1,2,\cdots ,q}{\left(x_{1}x_{2}\cdots x_{q}\right)}^{1/q}+\epsilon . \end{array}$$ (7)

In metallurgy when there are *q* = 2 ingredients in a mixture experiment, [18] found some polynomials were useful for modeling the response and called them Kasatkin’s polynomials. Such a polynomial of *n* ^*th*^ order has the form:

$$\begin{array}{c} y=\theta_{1}x_{1}+\theta_{2}x_{2}+\sum_{i=0}^{n-2}\phi_{i}x_{1}x_{2}{\left(x_{1}-x_{2}\right)}^{i}+\epsilon . \end{array}$$ (8)

Further details on rationale and applications of Becker’s and Kasatkin’s models can be found in [5], [18, 19], etc. Interestingly, these papers allude to *D*-optimal designs for Kasatkin’s polynomial models but we were unable to find the description of the *D*-optimal designs. In Section 4, we apply our modified PSO approach and generate *D*-optimal designs for Kasatkin’s polynomial models.

### Optimal Mixture Designs

[11], [20], [21], [22], [23], [24] and, [25] gave analytical descriptions of *D*-optimal designs for different orders of Scheffé polynomial models. Formulae for *A*- and integrated or *I*-optimal designs are available for a much smaller class of models.

[11] found the theoretical *A*- and *D*-optimal designs for the first order linear models with *q* factors over *S* ^*q*−1^. Both *A*- and *D*-optimal designs coincide and are equally supported on the *q* vertices of the simplex given by (1, 0, …, 0), …, (0, …, 0, 1). The *A*-optimal design for the quadratic mixture model with *q* ≥ 4 was found by [24] where they showed that the *A*-optimal design is the weighted {*q*, 2} simplex-centroid design. It has a combined weight of *r*_1_ = (4*q*−3)^1/2^/(*q*(4*q*−3)^1/2^ + 2*q*(*q*−1)) equally distributed among support points of the form (1, 0, …, 0), …, (0, …, 0, 1), and a combined weight of 4*r*_1_/(4*q*−3)^1/2^ equally distributed among points of the form (1/2, 1/2, 0, …, 0), …, (0, …, 0, 1/2, 1/2). When *q* = 3, they numerically identified the *A*-optimal as the weighted {*q*, 3} simplex-centroid design with (*r*_1_, *r*_2_, *r*_3_) = (0.1418, 0.1873, 0.0128), where *r*_1_, *r*_2_ are as before and *r*_3_ is now the weight at each of the support point of the form (1/3, 1/3, 1/3, 0 …, 0), …, (0, …, 0, 1/3, 1/3, 1/3).

We next consider two third-degree polynomial models for mixture studies; the first one does not incorporate a 3-way effect and is given by

$$\begin{array}{c} E\left(y\right)=\sum_{i=1}^{q}\beta_{i}x_{i}+\sum_{1\leq i<j\leq q}\beta_{ij}x_{i}x_{j}+\sum_{1\leq i<j\leq q+1}\gamma_{ij}x_{i}x_{j}\left(x_{i}-x_{j}\right). \end{array}$$ (9)

The *D*-optimal design was found to be equally supported at the the following design points: $C_{1}^{q+1}$ points given by *x*_*i*_ = 1, *x*_*j*_ = 0, *i* ≠ *j*, *i* = 1, …, *q*, $2C_{2}^{q+1}$ points given by $x_{i}=1-x_{j}=\frac{1}{2}(1-\frac{1}{\sqrt{5}}),\;\;i\neq j,i,j=1,\ldots ,q,$ and *x*_*k*_ = 0, *k* ≠ *i*, *j* [26]. The same author found the *D*-optimal design for the model

$$\begin{array}{c} E\left(y\right)=\sum_{i=1}^{q}\beta_{i}x_{i}+\sum_{1\leq i<j\leq q}\beta_{ij}x_{i}x_{j}+\sum_{1\leq i<j\leq q}\gamma_{ij}x_{i}x_{j}\left(x_{i}-x_{j}\right)+\sum_{1\leq i<j<k\leq q+1}\beta_{ijk}x_{i}x_{j}x_{k}. \end{array}$$ (10)

to be equally supported at the following design points: $C_{1}^{q+1}$ points given by *x*_*i*_ = 1, *x*_*j*_ = 0, *i* ≠ *j*, *i* = 1, …, *q*, $2C_{2}^{q+1}$ points given by $x_{i}=1-x_{j}=\frac{1}{2}(1-\frac{1}{\sqrt{5}}),\;\;i\neq j,i,j=1,\ldots ,q,$ *x*_*k*_ = 0, *k* ≠ *i*, *j*, and $C_{3}^{q+1}$ points given by *x*_*i*_ = *x*_*j*_ = *x*_*k*_ = 1/3, *x*_*l*_ = 0, *l* ≠ *i*, *j*, *k*; *i*, *j*, *k* = 1, 2, …, *q* + 1 [27].

An analytical description of the optimal design is desirable but as the above results show, they can be complicated even for relatively simple models and more frequently because of the mathematical complexity, they are usually not available. A more practical approach to find optimal designs is to use an algorithm. The next section describes a PSO-based algorithm that seems to have great potential for finding many types of optimal designs quickly for a variety of mixture and mixture-related models, including optimal designs for mixture models with constraints or on an irregular simplex or for some sub-models in Eq (10) for which analytical results remain elusive.

## Particle Swarm Optimization with Projection Capabilities

Particle swarm optimization (PSO), proposed by [28], is a general purpose optimization tool that can be generically and readily coded to simulate the behaviors of a flock of bird in search for food. PSO is a member of the class of nature-inspired meta-heuristic algorithms that has attracted a lot of attention in optimization research today [29, 30]. In its most basic form, PSO seeks to iteratively minimize a given function of several variables without requiring much of any assumption on the function. PSO works generically as follows. First, we specify the function Φ(**x**) to be optimized and the search space Ω. Second, we select a value of *N*, the flock size and initialize PSO by randomly generating *N* particles to search for the optimum over the search space. The particles represent candidates for the optimum solution. The two basic equations that drive movement for the particle *i* ^*th*^ in the PSO algorithm in its search to find the optimum is as follows. At times *t* and *t*+1, the movement of particle *i* is governed by the two equations

$$\begin{array}{c} v_{i}^{t+1}=w_{t}v_{i}^{t}+\gamma_{1}\alpha_{1}\left(p_{i}-x_{i}^{t}\right)+\gamma_{2}\alpha_{2}\left(p_{g}-x_{i}^{t}\right), \end{array}$$ (11)

and

$$\begin{array}{c} x_{i}^{t+1}=x_{i}^{t}+v_{i}^{t+1}. \end{array}$$ (12)

Here, ${\text{v}}_{i}^{t}$ and ${\text{x}}_{i}^{t}$ are, respectively, the velocity and the current position for the *i* ^*th*^ particle at time *t*. The inertia weight *w*_*t*_ modulates the influence of the former velocity and can be a constant or a decreasing function with values between 0 and 1. For example, [31] used a linearly decreasing function over the specified time range with an initial value 0.9 and end value of 0.4. The vector **p**_*i*_ is the personal best (optimal) position attained by the *i*th particle up to time *t* and the vector **p**_*g*_ is the global best (optimal) position attained among all particles up to time *t*. This means that up to time *t*, the personal best for particle *i* is *pbest*_*i*_ = Φ(**p**_*i*_) and *gbest* = Φ(**p**_*g*_). The two random vectors in the PSO algorithm are *α*_1_ and *α*_2_ and their components are usually taken to be independent random variables from *U*(0, 1). The constant *γ*_1_ is the cognitive learning factor and *γ*_2_ is the social learning factor. These two constants determine how each particle moves toward its own personal best position or overall global best position. The default values for these two constants in the PSO codes are *γ*_1_ = *γ*_2_ = 2 and they really seem to work well in practice for nearly all problems that we have investigated so far. Note that in Eq (11), the product in the last two terms is Hadamard product.

For our mixture design problem, the search space is the set of all approximate designs defined on the design space Ω, which is either a regular or irregular *q*-simplex. The optimality criterion Φ(*ξ*) is formulated as a convex function of the information matrix and our goal is to minimize Φ(*ξ*) over all approximate designs, *ξ*, on Ω. The initial flock of birds comprises randomly generated particles, which are design themselves, searching for the optimal mixture design. The particles are defined by their mass distributions and the support points, which is assumed to be the same in the whole flock. If the model has *d* parameters in the mean function, it is typical to choose the initial flock all with *d* support points. The above two equations define how each particle sequentially adapts its movement toward where it believes is the optimum and does so with an velocity that depends on its current location and locations that other particles believe is the optimum. The values of the parameters we used in the PSO are largely the default values described above. The function *w*_*t*_ we used for finding optimal designs for mixture experiments is the linear decreasing function that varies from 0.9 to 0.4.

Following convention, PSO can and should always be modified to take advantage the special features of the optimization problem at hand. For our mixture experiments design problems, we found that a more effective way to first optimize over the regular hypercube, and then use a projection function to identify our target optimal design in the search space Ω. To fix ideas, suppose the given mixture model has *q* factors and we wish to find a *k*-point optimal design. Let *m* = *k* × (*q* + 1) and let Ξ = [0, 1]^*m*^ denote the *m*-dimensional hypercube. Define the *m* × 1 vector, $\tilde{\xi }={\left({\text{x}}_{1}^{\prime },\ldots ,{\text{x}}_{k}^{\prime },{\text{p}}^{\prime }\right)}^{\prime }\in \Xi$, where **x**_*i*_ is a *q* × 1 vector in [0, 1]^*q*^, *i* = 1, …, *k*, **p** ∈ [0, 1]^*k*^ and define $\Xi^{*}=\Xi \backslash \{\tilde{\xi }={\left({\text{x}}_{1}^{\prime },\ldots ,{\text{x}}_{k}^{\prime },{\text{p}}^{\prime }\right)}^{\prime }\in \Xi \;|\;1_{k}^{\prime }\cdot \text{p}=0\;\text{or}\;1_{q}^{\prime }\cdot {\text{x}}_{i}=0\;\;\text{for some}\;i\}.$ To transform $\tilde{\xi }$ into a proper design *ξ*, we define the projection function *P*:Ξ* → (*S* ^*q*−1^)^*k*^ × *S* ^*k*−1^ by

$$\begin{array}{c} P\left(\tilde{\xi }\right)={\left(\frac{x_{1}^{{}^{\prime }}}{\left(1_{q}^{\prime }\cdot x_{1}\right)},\ldots ,\frac{x_{k}^{{}^{\prime }}}{\left(1_{q}^{\prime }\cdot x_{k}\right)},\frac{p^{\prime }}{\left(1_{k}^{\prime }\cdot p\right)}\right)}^{\prime }. \end{array}$$ (13)

The projection function *P* is invariant in the sense that $P\circ P(\tilde{\xi })=P(\tilde{\xi })$ and the design *ξ* has support on ${\tilde{\text{x}}}_{i}^{\prime }={\text{x}}_{i}^{\prime }/(1_{q}^{\prime }\cdot {\text{x}}_{i}),i=1,\ldots ,k$ and the components in ${\tilde{\text{p}}}^{\prime }={\text{p}}^{\prime }/(1_{k}^{\prime }\cdot \text{p})$ are the corresponding weights. The notation $\xi =P(\tilde{\xi })$ signifies that the design *ξ* is transformed from $\tilde{\xi }$ via the projection *P*.

Our modified PSO algorithm is based on the projection function *P* in Eq (13) as follows. We first initialize a random population of *n* candidates ${\tilde{\xi }}_{i}$ with *k* design points from Ξ*. We define two notions at each stage of the iteration: let (i) ${\tilde{\xi }}_{i}^{pbest}$ denote the personal best position for the *i* ^*th*^ particle, i.e. ${\tilde{\xi }}_{i}^{pbest}$ provides the optimal value for the criterion, $\Phi (\xi_{i})=\Phi (P({\tilde{\xi }}_{i}))$, among all the positions that the *i* ^*th*^ particle has ever visited, and (ii) let ${\tilde{\xi }}^{gbest}$ denote the global best position, i.e. *ξ* ^*gbest*^ provides the optimal value for the criterion among all the positions that all of the particles have ever visited. The strategy for the *i* ^*th*^ particle, ${\tilde{\xi }}_{i}$ at the *t* ^*th*^ iteration is as follows:

- Generate a new velocity ${\text{v}}_{i}^{t}$ to reach to next position given by ${\text{v}}_{i}^{t}=w_{t}{\text{v}}_{i}^{t-1}+\gamma_{1}\alpha_{1}({\tilde{\xi_{i}}}^{gbest}-{\tilde{\xi }}_{i}^{t-1})+\gamma_{2}\alpha_{2}({\tilde{\xi }}_{i}^{pbest}-{\tilde{\xi }}_{i}^{t-1}),$ where ${\text{v}}_{i}^{t-1}$ was the velocity used to get to the (*t*−1)^*th*^ iteration, *w*_*t*_ is the inertia weight, *γ*_1_, *γ*_2_ are two pre-specified positive constants, and *α*_1_, *α*_2_ are *m* × 1 uniform random vectors.
- The next location for the *i* ^*th*^ particle is
  $$\begin{array}{c} {\tilde{\xi }}_{i}^{t}={\tilde{\xi }}_{i}^{t-1}+v_{i}^{t}, \end{array}$$ (14)
  If ${\tilde{\xi }}_{i}^{t}$ is not in Ξ*, we project ${\tilde{\xi }}_{i}^{t}$ to a location closest to the boundary of Ξ*.
- Project ${\tilde{\xi }}_{i}^{t}$ onto the regular *q*-simplex using i.e. $\xi_{i}^{t}=P({\tilde{\xi_{i}}}^{t})$ and evaluate $\Phi (\xi_{i}^{t})$.
- Update the current best for each particle ${\tilde{\xi }}_{i}^{pbest}$.
- Update the inertia weight *w*_*t*+1_ = *g*(*w*_*t*_), where *g* is a user-selected monotonic decreasing function.

After updating all particles, ${\tilde{\xi }}_{i}^{t}$, we identify ${\tilde{\xi }}^{gbest}$, and repeat the procedure. When the procedure terminates, we project ${\tilde{\xi }}^{gbest}$ via the projection function *P* and report *ξ* ^*gbest*^ as our “best” design after a pre-specified maximal number of iterations is reached or when the criterion value does not change much according to some user-specified tolerance level.

The key advantage of this modified PSO algorithm is that it operates on the simple hypercube first before it projects any non-feasible point into (*S* ^*q*−1^)^*k*^ × *S* ^*k*−1^. This simplifies and makes the computation more efficient. If we had directly implemented PSO to search for the optimal mixture design, our experience is that some of the sequentially generated particles “flew” outside the simplex and the subsequent work required to ignore them or bring them back to the simplex can complicate and prolong the search for the best design considerably. Because we had expanded the search space from the simplex to the hypercube, multiple solutions can exist but PSO is able to handle problems with multiple solutions [32]. We call our proposed modified projection based PSO techniques ProjPSO and show in the next section that ProjPSO is effective in finding efficient designs for many types of mixture design problems.

## Numerical Results

In this section, we apply our ProjPSO algorithm to generate different types of optimal designs for different types of mixture experiment. We have verified that our algorithm produced the same optimal designs for many common models reported in the literature and for space consideration, we do not include them here. We focus on new optimal designs where analytical formulae for the optimal designs are not available or optimal designs that may be known but not commonly seen in the literature, such as those for Kasatkin’s polynomials. All ProjPSO-generated designs have been verified to be optimal using an equivalence theorem by plotting the directional derivative of the criterion at the ProjPSO-generated optimal designs over the design space. Figs 2 and 3 in Section 4 are examples of such plots.

We implemented the ProjPSO algorithm written in MATLAB codes in a PC with a 2.67GHz Intel(R) Core(TM) i7 CPU. All CPU times reported in this paper were based on this hardware. We always start with a modest size of the particles and a modest number of the iterations and increase them sequentially to expand the dimensionality of the experimental region as the model has more parameters. In almost all cases, ProjPSO was able to generate designs which were optimal or very close the theoretical optimal designs after a couple of minutes of CPU time. For example, to find the *D*-optimal design for the full cubic model with 3 factors, it took around 2.5 minutes to produce the analytical *D*-optimal design using 1024 particles and 200 iterations. Interestingly, for many of our problems, ProjPSO behaves like as reported in the literature in other disciplines in that it usually finds the optimal design in a mere few seconds of CPU time and spends the rest of time trying to get the optimal weights and support points agree to 4 or 5 decimal places.

**Fig 1:**
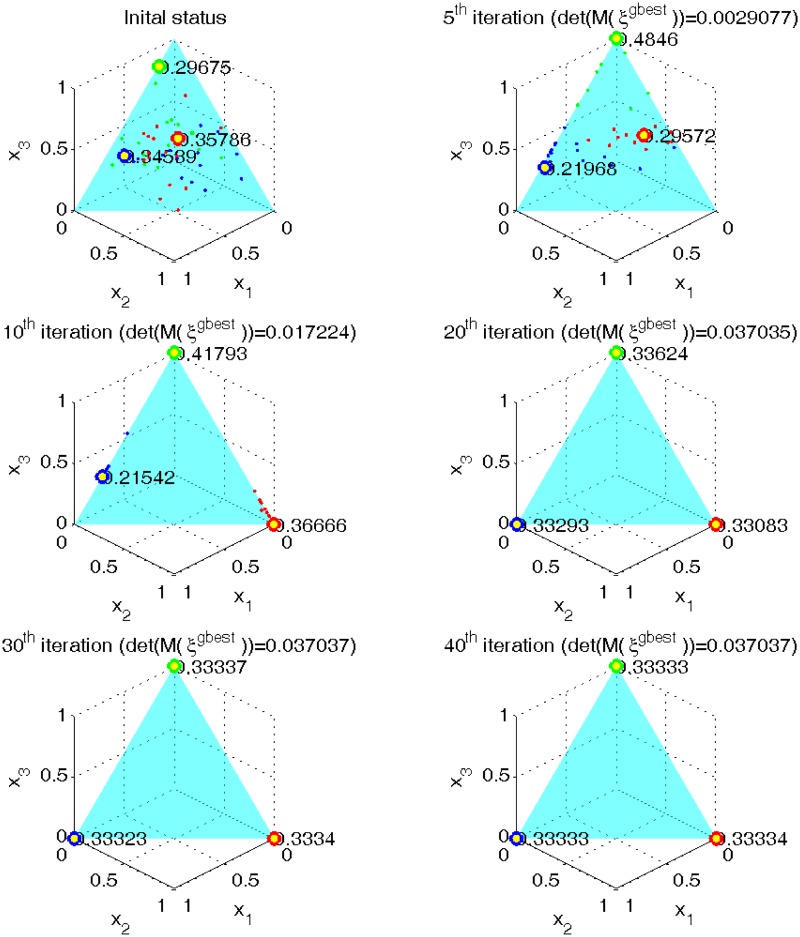
The movement of particles in the ProjPSO search for the *D*-optimal design for the linear additive mixture model with *q* = 3 factors. Each sub-figure displays the ProjPSO-generated mixture design or the global best design *ξ* ^*gbest*^ at a particular iteration. After 40 iterations and only 4 seconds of CPU time, ProjPSO converged to the *D*-optimal design equally supported at the vertices.

Fig 1 shows the movement of the particles at various stages in the search for the mixture linear additive model with *q* = 3 factors. Each particle represents a design defined by its location in the 3-dimensional plot whose 3 axes represent the range allowed for the 3 components in the mixture experiment. The mass distributions of the particles or designs at their support points are not shown (for clarity sake) except for 3 locations that the particles had quickly identified as potential support points of the optimal design. All particles have the same number of points. The sub-figures display the support points of the 64 particles at the 1^*st*^, 5^*th*^, 10^*th*^, 20^*th*^, 30^*th*^ and the 40^*th*^ iterations and show the very rapid convergence of the ProjPSO procedure to the optimum. The figures show that the *D*-optimal design was found roughly at the 30^*th*^ iteration and ProjPSO used 10 more iterations to ensure that the criterion value, each support point and each mass agree with their previous values up to 5 decimal places. After 3 seconds of CPU time, the ProjPSO algorithm generated a design equally supported at the 3 vertices, which is the theoretical optimal design.

In practice, we first searched using a flock where all have design points equal to the number of parameters in the model using the ProjPSO algorithm. When repeated number of times failed to produce the optimal design using different flock size and maximum number of iterations, this may suggest that the optimal design is not minimally supported. We then increase the number of support points of each design in the flock by one and repeat the process. Our guiding principle is larger number of particles or larger number of iterations for more complex models. Our experience is that the time required to generate the optimal design is usually fast and the difference in additional computational time required by either increasing the number of particles or iterations is usually not large. For instance, in the examples below, the number of particles we chose to generate the optimal designs for the linear Scheffé polynomial models were 64, 128 and 256 for *q* = 3, 4 and 5 and the corresponding number of iterations used were 200, 400 and 800.

We next applied ProjPSO to find the *A*- and *D*-optimal designs for Scheffé’s linear mixture models with *q* = 3, 4 and 5 factors. The ProjPSO-generated designs are all numerically the same as the theoretical *A*- and *D*-optimal designs reported in the literature. When we applied the ProjPSO algorithm to find *D*-optimal designs for the Scheffé’s quadratic mixture Eq (3), we also obtained the {*q*, 2} simplex-centroid design, which was already reported by Kiefer to be *D*-optimal [20].

We were also able to verify ProjPSO-generated designs for Scheffé’s cubic model with and without 3-way effects are the same as those reported in [26, 27]. In addition, we modified ProjPSO codes to find *I*-optimal designs for the Scheffé’s quadratic and cubic mixture models using 1024 particles and 400 iterations. An equivalence theorem was used to confirm that the ProjPSO-generated design was optimal in each case and it is the same as the ones reported in the literature. The next few subsections present optimal designs or nearly optimal designs that we have obtained using ProjPSO for different mixture models under various setups. They are either new results or hard to find optimal designs reported in the literature, such as those for Kasatkin’s polynomials.

### Incomplete Scheffé’s Models

**Fig 2:**
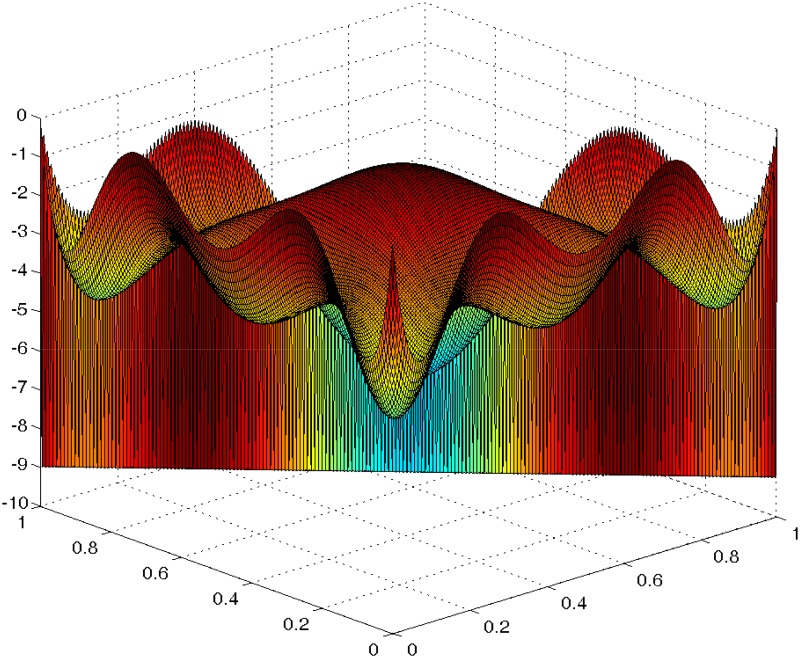
The plot of the directional derivative of the ProjPSO-generated design confirms it is *D*-optimal design for the IC Model A.

We also used ProjPSO to determine optimal designs for several submodels obtained by deleting a few interaction terms from the full cubic model. These submodels or incomplete (IC) models are less studied even though they have been used in the development and optimization of microemulsion formulations in mixture experiments in the pharmaceutical industry [4]. As far as we know, theoretical optimal designs remain unknown for these models. We applied ProjPSo with 1024 particles and iterated 400 times to find *D*-optimal designs for these models. To ensure the generated designs are *D*-optimal, we used equivalence theorems to confirm their optimality. Fig 2 shows the directional derivative of the criterion evaluated at the optimum for the submodel IC Model A in Table 1. The 3-dimensional plot is bounded above by 0 with equality at the support points of the ProjPSO-generated design and so the optimality of the reported design in Table 1 is confirmed.

**Table 1:** ProjPSO-generated *D*-optimal designs for submodels from Scheffé polynomial models with 3 factors.

model	type	support points	weights
IC Model A	∑_*i*_ *β*_*i*_ *x*_*i*_+*β*_13_ *x*_1_ *x*_3_+*β*_23_ *x*_2_ *x*_3_+∑_*i*<*j*_ *γ*_*ij*_ *x*_*i*_ *x*_*j*_(*x*_*i*_−*x*_*j*_)+*β*_123_ *x*_1_ *x*_2_ *x*_3_	(1, 0, 0), (0, 1, 0)	0.0833 ×2
		(0, 0, 1)	0.1111 ×1
		(0.2764, 0.7236, 0), (0.2764, 0, 0.7236)	0.1111 ×4
		(0.2113, 0.7887, 0), (0.7887, 0.2113, 0)	0.0833 ×2
		(0.3333, 0.3333, 0.3333)	0.1111 ×1
IC Model B	∑_*i*_ *β*_*i*_ *x*_*i*_+*β*_23_ *x*_2_ *x*_3_+∑_*i*<*j*_ *γ*_*ij*_ *x*_*i*_ *x*_*j*_(*x*_*i*_−*x*_*j*_)+*β*_123_ *x*_1_ *x*_2_ *x*_3_	(1, 0, 0)	0.0938 ×3
		(0, 0.2764, 0.7236), (0, 0.7236, 0.2764)	0.1250 ×2
		(0.7887, 0.2113, 0), (0.7887, 0, 0.2113)	0.0937 ×2
		(0.2113, 0.7887, 0), (0.2113, 0, 0.7887)	0.0937 ×2
		(0.3333, 0.3333, 0.3333)	0.1250 ×1
IC Model C	∑_*i*_ *β*_*i*_ *x*_*i*_+∑_*i*<*j*_ *β*_*ij*_ *x*_*i*_ *x*_*j*_+*γ*_13_ *x*_1_ *x*_3_(*x*_1_−*x*_3_)+*γ*_23_ *x*_2_ *x*_3_(*x*_2_−*x*_3_)	(1, 0, 0)	0.1250 ×3
		(0, 0.2764, 0.7236), (0.2764, 0, 0.7236)	0.1250 ×2
		(0.7236, 0, 0.2764), (0, 0.7236, 0.2764)	0.1250 ×2
		(0.5, 0.5, 0))	0.1250 ×1
IC Model D	∑_*i*_ *β*_*i*_ *x*_*i*_+∑_*i*<*j*_ *β*_*ij*_ *x*_*i*_ *x*_*j*_+*γ*_13_ *x*_1_ *x*_3_(*x*_1_−*x*_3_)+*γ*_23_ *x*_2_ *x*_3_(*x*_2_−*x*_3_)+*β*_123_ *x*_1_ *x*_2_ *x*_3_	(1, 0, 0)	0.1111 ×3
		(0, 0.2764, 0.7236), (0.2764, 0, 0.7236)	0.1111 ×2
		(0.7236, 0, 0.2764), (0, 0.7236, 0.2764)	0.1111 ×2
		(0.5, 0.5, 0))	0.1111 ×1
		(0.3333, 0.3333, 0.3333)	0.1111 ×1

### Optimal Designs for Becker’s and Kasatkin’s Models

We next apply ProjPSO to generate *D*-optimal designs for other mixture models to demonstrate its flexibility. For illustrative purposes, we consider 3 Becker’s models shown in Eqs (5)–(7) with *q* = 3 and the Kasatkin’s polynomial models of order 3, 4, and 5 in Eq (8). In our ProjPSO code, we only need to change the regressor set-up for the target model. PSO found the numerical best designs for all 6 models and all were verified that they satisfied the equivalence theorems. Table 2 shows the *D*-optimal designs for these models.

**Table 2:** ProjPSO-generated *D*-optimal designs for Becker and Kasatkin’s mixture models with 3 factors.

model	type	support points	weights
Becker Model 1 Becker (1968, 1978)	∑_*i*_ *β*_*i*_ *x*_*i*_+∑_*i*<*j*_ *β*_*ij*_(*x*_*i*_ *x*_*j*_)^(1/2)^+*β*_123_(*x*_1_ *x*_2_ *x*_3_)^(1/3)^	(1, 0, 0)	0.1429 ×3
		(0.5, 0.5, 0)	0.1429 ×3
		(0.3333, 0.3333, 0.3333)	0.1429 ×1
Becker Model 2 Becker (1968, 1978)	∑_*i*_ *β*_*i*_ *x*_*i*_+*β*_12_ *x*_1_ *x*_2_/(*x*_1_+*x*_2_)+*β*_13_ *x*_1_ *x*_3_/(*x*_1_+*x*_3_)+*β*_23_ *x*_2_ *x*_3_/(*x*_2_+*x*_3_)+*β*_123_ *x*_1_ *x*_2_ *x*_3_	(1, 0, 0)	0.1429 ×3
		(0.5, 0.5, 0)	0.1429 ×3
		(0.3333, 0.3333, 0.3333)	0.1429 ×1
Becker Model 3 Becker (1968, 1978)	∑_*i*_ *β*_*i*_ *x*_*i*_+*β*_12_min{*x*_1_, *x*_2_}+*β*_13_min{*x*_1_, *x*_3_}*β*_23_min{*x*_2_, *x*_3_}+*β*_123_min{*x*_1_, *x*_2_, *x*_3_}	(1, 0, 0)	0.1429 ×3
		(0.5, 0.5, 0)	0.1429 ×3
		(0.3333, 0.3333, 0.3333)	0.1429 ×1
Kasatkin 3rd Order Model Kasatkin (1974)	$\sum_{i=1}^{2}\theta_{i}x_{i}+\sum_{i=0}^{1}\phi_{i}x_{1}x_{2}{\left(x_{1}-x_{2}\right)}^{i}$	(1, 0, 0), (0, 1, 0)	1/4 ×2
		(0.2764, 0.7236, 0), (0.7236, 0.2764, 0)	1/4 ×2
Kasatkin 4th Order Model Kasatkin (1974)	$\sum_{i=1}^{2}\theta_{i}x_{i}+\sum_{i=0}^{2}\phi_{i}x_{1}x_{2}{\left(x_{1}-x_{2}\right)}^{i}$	(1, 0, 0), (0, 1, 0)	1/5 ×2
		(0.1727, 0.8273, 0), (0.8273, 0.1727, 0)	1/5 ×2
		(0.5, 0.5, 0)	1/5
Kasatkin 5th Order Model Kasatkin (1974)	$\sum_{i=1}^{2}\theta_{i}x_{i}+\sum_{i=0}^{3}\phi_{i}x_{1}x_{2}{\left(x_{1}-x_{2}\right)}^{i}$	(1, 0, 0), (0, 1, 0)	1/6 ×2
		(0.1175, 0.8825, 0), (0.8825, 0.1175, 0)	1/6 ×2
		(0.3574, 0.6426, 0), (0.6426, 0.3574, 0)	1/6 ×2

### Mixture Models with Many Factors

To test the capability of our algorithm ProjPSO, we applied it to mixture models with many factors. For this purpose, we consider Scheffé linear mixture model with *q* = 10 and *q* = 20 factors and Scheffé quadratic mixture model with *q* = 6 and *q* = 8 factors. We assumed for these models conservatively that the optimal designs are supported at a minimum number of points. This means the number of support points of the optimal design is equal to the number of parameters in the model; if we had fewer points, the information matrix if the design will be singular.

For the linear model with 10 parameters, the number *m* of variables to optimize in our optimization problem is *m* = 10 × (10 + 1) = 110. Using 1024 particles and 400 iterations in our ProjPSO, the ProjPSO-generated *D*-optimal design was uniformly supported at *e*_*i*_, *i* = 1, …, 10, where *e*_*i*_ is the 0 vector except its *i* ^*th*^ component is equal to unity. With *q* = 20 factors, the number of variables to be optimized in the linear model is now *m* = 20 × (20 + 1) = 420. We applied ProjPSO and used an initial flock size with 2048 particles and after 400 iterations, ProjPSO was able to correctly identified the theoretical *D*-optimal design uniformly supported at *e*_*i*_, *i* = 1, …, 20.

We next apply ProjPSO to find a *D*-optimal design for the Scheffé quadratic model with *q* = 6 and *q* = 8 factors. This means that there are 21 parameters in the first model and 36 parameters in the second model. If the *D*-optimal designs are minimally supported, the dimensions of the optimization problems that ProjPSO has to solve are *m* = 21 × (6 + 1) = 147 and *m* = 36 × (8 + 1) = 324 respectively. For the first problem, we applied ProjPSO with 10240 particles and 1000 iterations and the *D*-optimal design found by ProjPSO had 6 vertices with one run at each of the midpoints of the 15 edges of the tetrahedral mixture simplex region. In the second problem, we applied ProjPSO with 10240 particles and 1500 iterations. Convergence was not attained at the end of the 1500 iterations but over repeated runs, the highest efficiency obtained was 0.9985. When we increased the number of particles to 66560 particles, the efficiency of the best design produced by ProjPSO after 1500 iterations was 0.9999, which is optimal for all practical purposes. The generated design was equally supported at the 8 vertices with one run at each of the midpoints of the 28 edges of the tetrahedral mixture simplex region.

### The Linear Log Contrast Models

This subsection shows ProjPSO can be directly modified to find optimal designs for the linear log contrast models proposed by [33]. [34] found the *D*-optimal approximate design for the log contrast model given by

$$\begin{array}{c} E\left(y\right)=\beta_{0}+\sum_{i=1}^{q-1}\beta_{i}\log\left(x_{i}/x_{q}\right). \end{array}$$

Recent design work for the linear log contrast model includes [12] and, [13] who found exact *D*- and *A*-optimal designs for linear log contrast and quadratic log contrast models. To ensure a *D*-optimal design for such a model exists, additional constraints on all the factors are required. One common way to do this is to select a constant *δ* ∈ (0, 1) with the conditions *δ* ≤ *x*_*i*_/*x*_*j*_ ≤ 1/*δ*, for all 1 ≤ *i*, *j* ≤ *q* as added constraints on the design region *S* ^*q*−1^.

As an illustration, consider the log contrast model with *q* = 3. [34] showed that for a given *δ*, the *D*-optimal design has 3 points and is supported equally at (1/(1+2*δ*), *δ*/(1+2*δ*), *δ*/(1+2*δ*)), (*δ*/(1+2*δ*), 1/(1+2*δ*), *δ*/(1+2*δ*)), (*δ*/(1+2*δ*), *δ*/(1+2*δ*), 1/(1+2*δ*)), or (1/(2+*δ*), 1/(2+*δ*), *δ*/(2+*δ*)), (*δ*/(2+*δ*), 1/(2+*δ*), 1/(2+*δ*)), (1/(2+*δ*), *δ*/(2+*δ*), 1/(2+*δ*)).

To find the optimal design using ProjPSO, we redefined the regressors as log(*x*_*i*_/*x*_*q*_) and also amended the projection operator in ProjPSO so that it projects into the right space that includes the additional constraints, *δ* ≤ *x*_*i*_/*x*_*j*_ ≤ 1/*δ* for all *i*. We used ProjPSO to find the D-optimal designs when *δ* = 0.145 and 0.2. Using a flock size of 1024 and 100 number of iterations, ProjPSO took approximately 11 seconds of CPU time to generate the *D*-optimal designs below, which also agree with the result in [34]. For each of these two *δ*’s, there are two optimal designs equally supported at 3 points. For *δ* = 0.145, one set of support points is ${\tilde{\text{x}}}_{1}^{\prime }=(0.1124,0.1124,0.7752)$, ${\tilde{\text{x}}}_{2}^{\prime }=(0.7752,0.1124,0.1124)$ and ${\tilde{\text{x}}}_{3}^{\prime }=(0.1124,0.1124,0.7752)$ and the other set is ${\tilde{\text{x}}}_{1}^{\prime }=(0.4662,0.4662,0.0676)$, ${\tilde{\text{x}}}_{2}^{\prime }=(0.0676,0.4662,0.4662)$ and ${\tilde{\text{x}}}_{3}^{\prime }=(0.4662,0.0676,0.4662).$ For *δ* = 0.2, one set of support points is ${\tilde{\text{x}}}_{1}^{\prime }=(0.7143,0.1429,0.1429)$, ${\tilde{\text{x}}}_{2}^{\prime }=(0.1429,0.7143,0.1429)$ and ${\tilde{\text{x}}}_{3}^{\prime }=(0.1429,0.1429,0.7143)$ and the other set of support points is ${\tilde{\text{x}}}_{1}^{\prime }=(0.0909,0.4545,0.4545)$, ${\tilde{\text{x}}}_{2}^{\prime }=(0.4545,0.0909,0.4545)$ and ${\tilde{\text{x}}}_{3}^{\prime }=(0.4545,0.4545,0.0909).$

We note here that this is one situation where we did not obtain good results using the default values *γ*_1_ = *γ*_2_ = 2 in the ProjPSO algorithm. This may be due to the smaller design space resulting from the several constraints. Our experience suggests that setting *γ*_1_ = *γ*_2_ = 0.5 seems to work well for log contrast models.

### Mixture Problems with Variable Constraints

Mixture experiments sometimes have physical constraints imposed on the components. Because of practical or cost considerations, upper or lower bound constraints are imposed on some of the *x*_*i*_’s with user-specified constants *L*_*i*_ and *U*_*i*_, such that *L*_*i*_ ≤ *x*_*i*_ ≤ *U*_*i*_, *i* = 1, 2, …, *q*. Examples where mixture experiments have constraints on the components abound in pharmaceutical problems as well. For instance in tablet formulations, typically a *D*-optimal design is sought in the constrained mixture design with limits imposed on the various ingredients, see for example [3, 35, 36] and [37]. In what is to follow, we directly modified the ProjPSO algorithm to find an efficient design for 2 applications to estimate parameters in the mixture model for such studies by including these constrains into our optimization problems.

The first example concerns a cubic mixture model without the 3-way interaction term and there is a constraint on the percent of the first component in the mixture being not larger than one half, i.e. *x*_1_ ≤ 0.5. The mean function is the cubic model in Eq (9) i.e. *q* = 3. To find the *D*-optimal design, we modified ProjPSO and found a 9-point optimal design using 1024 particles and 1000 iterations. This design $\xi_{constrained,D}^{3}$ is equally supported at 9 support points ${\tilde{\text{x}}}_{i}^{\prime }=(x_{i1},x_{i2},x_{i3})$ shown below:

$$\begin{array}{ccccccccc} {\tilde{x}}_{1} & {\tilde{x}}_{2} & {\tilde{x}}_{3} & {\tilde{x}}_{4} & {\tilde{x}}_{5} & {\tilde{x}}_{6} & {\tilde{x}}_{7} & {\tilde{x}}_{8} & {\tilde{x}}_{9}\\ 0.5000 & 0.3645 & 0.0000 & 0.2135 & 0.5000 & 0.0000 & 0.0000 & 0.2135 & 0.0000\\ 0.5000 & 0.3178 & 0.0000 & 0.7865 & 0.0000 & 1.0000 & 0.2764 & 0.0000 & 0.7236\\ 0.0000 & 0.3178 & 1.0000 & 0.0000 & 0.5000 & 0.0000 & 0.7236 & 0.7865 & 0.2764 \end{array}$$

**Fig 3:**
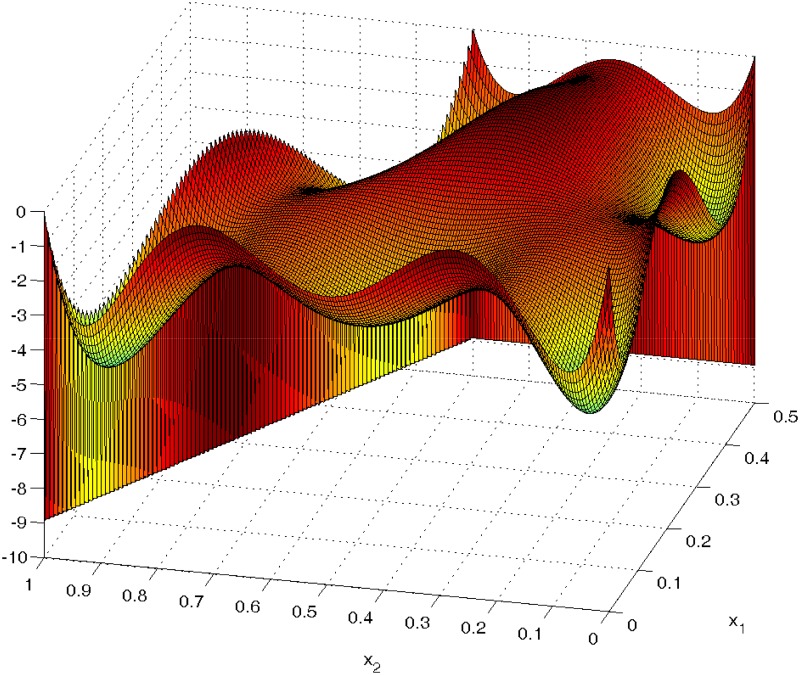
The plot of the directional derivative of the ProjPSO-generated design confirms that $\xi_{constrained,D}^{3}$ is *D*-optimal design for the cubic mixture model without the 3-way interaction term on the irregular 3-simplex with $\sum_{i=1}^{3}x_{i}=1$, 0 ≤ *x*_1_ ≤ 0.5 and 0 ≤ *x*_2_, *x*_3_ ≤ 1.

Fig 3 is the plot of the directional derivative of this generated design $\xi_{constrained,D}^{3}$ given by $f{\left(x\right)}^{\prime }M^{-1}(\xi_{constrained,D}^{3})f(x)-9$. It shows that the derivative is always bounded above by 0 with equality at the support points and so confirms the *D*-optimality of this design.

The goal of our second example is to further test the ability of ProjPSO to find a *D*-optimal design for the Scheffé linear mixture model with 6 factors and each component *x*_*i*_ is restricted by 0 ≤ *x*_*i*_ ≤ *a*_*i*_ and the *a*_*i*_’s are independently drawn from *Unif*[0.5, 1]. For our example, the *a*_*i*_’s are 0.6133, 0.8572, 0.5478, 0.8094, 0.5075 and 0.6871. We employed ProjPSO with 15,360 particles and 6000 iterations to search for the *D*-optimal design over the irregular simplex region. The ProjPSO-generated design has unequal weights at 13 points given by

$$\begin{array}{ccccccccccccc} {\tilde{x}}_{1} & {\tilde{x}}_{2} & {\tilde{x}}_{3} & {\tilde{x}}_{4} & {\tilde{x}}_{5} & {\tilde{x}}_{6} & {\tilde{x}}_{7} & {\tilde{x}}_{8} & {\tilde{x}}_{9} & {\tilde{x}}_{10} & {\tilde{x}}_{11} & {\tilde{x}}_{12} & {\tilde{x}}_{13}\\ 30.0000 & 0.0000 & 0.1906 & 0.0000 & 0.0000 & 0.4522 & 0.4925 & 0.6133 & 0.0000 & 0.0000 & 0.6131 & 0.0000 & 0.1428\\ 0.0000 & 0.0000 & 0.0000 & 0.0000 & 0.4925 & 0.0000 & 0.0000 & 0.0000 & 0.0000 & 0.0000 & 0.0000 & 0.8572 & 0.8572\\ 0.3129 & 0.0000 & 0.0000 & 0.0000 & 0.0000 & 0.5478 & 0.0000 & 0.0000 & 0.5439 & 0.0000 & 0.0000 & 0.1428 & 0.0000\\ 0.0000 & 0.8094 & 0.8094 & 0.0000 & 0.0000 & 0.0000 & 0.0000 & 0.0000 & 0.0000 & 0.8094 & 0.3869 & 0.0000 & 0.0000\\ 0.0000 & 0.1906 & 0.0000 & 0.3129 & 0.5075 & 0.0000 & 0.5075 & 0.0000 & 0.0000 & 0.0000 & 0.0000 & 0.0000 & 0.0000\\ 0.6871 & 0.0000 & 0.0000 & 0.6871 & 0.0000 & 0.0000 & 0.0000 & 0.3867 & 0.4561 & 0.1906 & 0.0000 & 0.0000 & 0.0000 \end{array}$$

The corresponding weights at these points are 0.0885, 0.0825, 0.0208, 0.0931, 0.0325, 0.0988, 0.1005, 0.0783, 0.0007, 0.0553, 0.0332, 0.0845 and 0.0781. The *D*-efficiency lower bound of the above design can be directly calculated to be 0.9701 implying that the ProjPSO-generated design is close to the *D*-optimal design and likely suffice for all practical purposes.

## Comparative Performance of ProjPSO

There are several algorithms for finding optimal designs for mixture experiments. Early algorithms were quite comprehensively reviewed in [38] and they include Dykstra’s method [39], Wynn-Mitchell’s method [40, 41], DETMAX [42] and the modified Fedorov’s methods.

In this section, we compared results from the ProjPSO techniques with a few popular or recent methods for finding optimal designs for mixture models. We first discuss the various methods in the following order: (a) the OPTEX procedure in the commercial software package SAS, (b) the AlgDesign package in the free software R and (c) two recent modified exchange types of algorithms, one called the Cocktail algorithm proposed by [43] and the other proposed by [1] and referred to as the YBT algorithm, after the initials of the last names of the authors. The latter two may be considered state-of-the-art algorithms for finding *D*-optimal designs. Comparisons were made for each of the 4 methods using various models but because of space constraint, we only report selected but representative results from our work. We note that the OPTEX procedure is for finding exact optimal designs and the rest are for finding approximate optimal designs. Our last example shows ProjPSO can also be used to find multistage designs as described in [1]. Unlike ProjPSO techniques, all 4 algorithms require that the design space be discretized using a user-selected grid set. The grid is usually formed by having a fixed number of uniformly space points over the design space for each factor.

(a) Comparison with the OPTEX procedure in SAS:

Commerical statistical software packages like SAS and JMP typically have a few menus for finding optimal designs for multi-factor polynomial models and mixture models, such as Scheffé polynomial models. However, these packages usually are available for searching exact *D*-optimal designs and sometimes also for exact *A* and *I*-optimal designs optimal designs. Different packages employ different methods for finding optimal designs. For instance, SAS uses the exchange coordinate type algorithms and JMP uses the candidate-free exchange algorithm. When a model of interest is not available in the package, it is not always clear if the program provides a way to find the optimal design for the model of interest. For instance, we were unable to find a statistical package capable of directly generating the *D*-optimal designs in Table 2.

To compare the *D*-optimal designs generated from OPTEX procedure in SAS, we consider as an example, the incomplete 3-factor cubic Scheffé’s polynomials given by

$$\begin{array}{c} E\left(y\right)=\beta_{0}+\beta_{1}x_{1}+\beta_{2}x_{2}+\beta_{11}x_{1}^{2}+\beta_{12}x_{1}x_{2}+\beta_{22}x_{2}^{2}+\beta_{111}x_{1}^{3}+\beta_{122}x_{1}x_{2}^{2}. \end{array}$$ (15)

We applied ProjPSO with 1024 particles and 400 iterations to find the *D*-optimal design. The generated design *ξ*_*PSO*−*D*_ is

$$\begin{array}{ccccccccc} {\tilde{x}}_{1} & {\tilde{x}}_{2} & {\tilde{x}}_{3} & {\tilde{x}}_{4} & {\tilde{x}}_{5} & {\tilde{x}}_{6} & {\tilde{x}}_{7} & {\tilde{x}}_{8} & {\tilde{x}}_{9}\\ 0.0000 & 0.0000 & 0.2774 & 0.0000 & 0.2723 & 0.6991 & 0.7238 & 0.2767 & 1.0000\\ 0.0000 & 1.0000 & 0.2491 & 0.4946 & 0.0000 & 0.0000 & 0.2762 & 0.7233 & 0.0000\\ 1.0000 & 0.0000 & 0.4735 & 0.5054 & 0.7277 & 0.3009 & 0.0000 & 0.0000 & 0.0000 \end{array}$$

and the weight vector is (0.1198, 0.1249, 0.0802, 0.1182, 0.0647, 0.1212, 0.1210, 0.1250, 0.1250). A plot based on the equivalence theorem confirmed its optimality.

The OPTEX procedure in SAS uses the modified Fedorov’s exchange algorithm to generate *D*-optimal designs after we pre-specify the number of runs and a grid set. Based on the above design, we used a grid set with 101 points uniformly spread out for each factor and applied the OPTEX procedure to find a 9–point exact *D*-optimal design. SAS produced the following equally-weighted design *ξ*_*SAS*−*D*_ after 1000 iterations:

$$\begin{array}{ccccccccc} {\tilde{x}}_{1} & {\tilde{x}}_{2} & {\tilde{x}}_{3} & {\tilde{x}}_{4} & {\tilde{x}}_{5} & {\tilde{x}}_{6} & {\tilde{x}}_{7} & {\tilde{x}}_{8} & {\tilde{x}}_{9}\\ 0.0000 & 0.0000 & 0.0000 & 0.2700 & 0.2800 & 0.2800 & 0.7000 & 0.7300 & 1.0000\\ 0.0000 & 0.4900 & 1.0000 & 0.2900 & 0.0000 & 0.7200 & 0.0000 & 0.2700 & 0.0000\\ 1.0000 & 0.5100 & 0.0000 & 0.4400 & 0.7200 & 0.0000 & 0.3000 & 0.0000 & 0.0000 \end{array}$$

The relative *D*-efficiency of the two designs is {*det*(*M*(*ξ*_*SAS*−*D*_))/*det*(*M*(*ξ*_*PSO*−*D*_))}^1/8^ = 0.9841, implying that the ProjPSO-generated design *ξ*_*PSO*−*D*_ is more efficient. The ProjPSO-generated design continue to outperform the SAS-generated *D*-optimal designs when we wanted a 18-point or a 27-point design. In the former case, the relative efficiency of the two designs was 0.9887, and in the latter case, the relative efficiency was 0.9938. This suggests that as the sample size increases, the SAS-generated designs are increasingly more efficient relative to *ξ*_*PSO*−*D*_ as expected.

(b) Comparison with the AlgDesign package in R:

We next compare performance of the algorithm called AlgDesign in the free software R package for generating optimal approximate designs for mixture models. This package uses the Federov exchange algorithm under the name optFederov to calculate approximate designs for the *D*-, *A*- and *I*-criteria. The algorithm quits when no more profitable exchanges are possible. Optimal designs can be generated using the function gen.mixture and the function “optFederov” after a candidate set of design points is pre-specified to search for the design points in the optimal design. Further details of the algorithm can be found at website http://cran.r-project.org/web/packages/AlgDesign/index.html.

We implemented the AlgDesign procedure using a grid set with 100 points uniformly spread out for each factor. Results found from AlgDeisgn and our ProjPSO algorithm were basically the same but we observed optimal designs found from the latter are sometimes slightly better in terms of the criterion value. For example, for the full cubic model with 3 factors, the optimal design *ξ*_*AD*−*D*_ found by AlgDesign had 33 design points whereas the one found by ProjPSO *ξ*_*PSO*−*D*_ had 10 points. The relative *D*-efficiency of the two designs was {*det*(*M*(*ξ*_*AD*−*D*_))/*det*(*M*(*ξ*_*PSO*−*D*_))}^1/10^ = 0.9985. As another example, for the quadratic model with 4 factors, AlgDesign produced a 25-point *A*-optimal design and ProjPSO produced a design with only 10 points. The *A*-efficiency of the AlgDesign produced design relative to the ProjPSO produced design is *trace* *M*(*ξ*_*PSO*−*A*_)^−1^/*trace* *M*(*ξ*_*AD*−*A*_)^−1^ = 0.9668. In either case, the ProjPSO-generated design wins.

(c) Comparison with two new modified exchange type algorithms:

Recently, two state-of-the-art algorithms for finding *D*-optima designs were proposed in [43] and [1]. [43] proposed the “Cocktail” algorithm to generate approximate *D*-optimal designs by combining the vertex direction method and the multiplicative algorithm. To improve the computational efficiency, a new nearest neighbor exchange strategy is adopted. To implement this Cocktail algorithm, we also need to discretize the design space first. Suppose the grid set has *r* candidate points and *w* = (*w*_1_, …, *w*_*r*_) is the probability vector for all these points as potential support points of the optimal designs. Clearly, $\sum_{i=1}^{r}w_{i}=1$ and the Cocktail algorithm optimizes the *D*-optimality criterion iteratively with respect to *w*. The stopping criterion is based on the equivalence theorem for *D*-optimal design and the algorithm terminates when the generated design *ξ* satisfies $\frac{1}{m}{\text{max}}_{i}f{\left({\text{x}}_{i}\right)}^{⊤}M^{-1}(\xi )f({\text{x}}_{i})\leq 1+ɛ,$ where *m* is the number of the coefficients in the model, **x**_*i*_ is the *i*th candidate point, and *ɛ* is the pre-specified tolerance level.

We obtained the MATLAB code for the Cocktail algorithm from Yaming Yu’s web-site and recoded it using the weights exchange algorithm to search for *D*-optimal designs for the mixture Eq (15). The grid set was uniformly spaced across the simplex with 1001 grid points for each factor, resulting in a total of 501,501 points. We used the default set-up of this Cocktail MATLAB code with *ɛ* = 10^−6^ and 127.4690 seconds of CPU time, the algorithm stopped at the 42^*th*^ iteration. The Cocktail algorithm generated design *ξ*_*Cocktail*−*D*_ has 10 points at

$$\begin{array}{cccccccccc} {\tilde{x}}_{1} & {\tilde{x}}_{2} & {\tilde{x}}_{3} & {\tilde{x}}_{4} & {\tilde{x}}_{5} & {\tilde{x}}_{6} & {\tilde{x}}_{7} & {\tilde{x}}_{8} & {\tilde{x}}_{9} & {\tilde{x}}_{10}\\ 0.0000 & 0.0000 & 0.0000 & 0.2720 & 0.2770 & 0.2770 & 0.2780 & 0.6990 & 0.7240 & 1.0000\\ 0.0000 & 0.4950 & 1.0000 & 0.0000 & 0.2490 & 0.7230 & 0.2490 & 0.0000 & 0.2760 & 0.0000\\ 1.0000 & 0.5050 & 0.0000 & 0.7280 & 0.4740 & 0.0000 & 0.4730 & 0.3010 & 0.0000 & 0.0000 \end{array}$$

and the weight distribution vector is (0.1198, 0.1182, 0.1249, 0.0647, 0.0566, 0.1250, 0.0236, 0.1212, 0.1210, 0.1250). The *D*-efficiency of the this design relative to *ξ*_*PSO*−*D*_ is {*det*(*M*(*ξ*_*Cocktail*−*D*_))/*det*(*M*(*ξ*_*PSO*−*D*_))}^1/8^ = 1.0000 and so both designs are very close in terms of the *D*-optimality criterion. our ProjPSO code with 1024 particles and 400 iterations took 57.3281 seconds which is also fewer than that required from the Cocktail MATLAB code.

[1] proposed an algorithm to generate optimal designs for a broad class of design criteria that include *D* and *c*-optimality. This YBT algorithm also requires a user-specified grid before it uses an exchange type approach to optimize the weights of the current design points via the Newton-Raphson method. The points with zero weight are dropped and the new design point to be added is the one that maximizes the directional derivative of the objective function. Thus their algorithm is also sometimes called “optimal weights exchange algorithm”.

The stopping criterion for the YBT algorithm is based on the maximal values of the directional derivative of the objective function at all the design points. Following [1], we recoded the SAS code provided by Yang on his website into MATLAB code and ran the code using the same tolerance level in [1] with *ɛ* = 10^−6^. After 206.225 seconds, the best design found by the YBT algorithm is the same as *ξ*_*Cocktail*−*D*_. Table 3 shows the design points, the relative *D*-efficiencies and CPU time for these two algorithms using different grid sizes.

**Table 3:** Number of support points in the generated designs from the Cocktail and Optimal Weights Exchange algorithms using different grid sizes, their relative *D*-efficiencies and CPU times for the model in Eq (15).

Grid size	101	501	1001	2001
CA	12 points, 0.9999, 1.15625 secs	12 points, 1.0000, 43.4063 secs	10 points, 1.0000, 125.219 secs	10points, 1.0000, 648.922 secs
Yang	12 points, 0.9999, 4.988 secs	12 points, 1.0000, 141.232 secs	10 points, 1.0000, 206.225 secs	12 points, 1.0000, 1250.920 secs

It is clear from the tables that the OPTEX procedure and the two new exchange algorithms can quickly produce highly efficient designs when we use a small grid size to search for the *D*-optimal design. However, they almost never are able to find the *D*-optimal designs because by construction they are dependent on the grid size employed. Further, optimal designs produced from the Cocktail and the YBT algorithms typically have more design points than are needed. For example, the design *ξ*_*Cocktail*−*D*_ has one pair of points, ${\tilde{\text{x}}}_{5}$ and ${\tilde{\text{x}}}_{7}$, that one imagines will be correctly merged into one design point with a much finer grid and longer computational time.

The above comparisons were carried out using the Scheffe polynomial Eq (15). Other mixture models we looked at produced similar results. For example, we found corresponding results for IC models A and B in Table 1 and they appear similar to those for Eq (15) and so are omitted for space consideration. All 4 algorithms can quickly generate highly efficient designs which have slightly more design points than are needed but the additional points merged as the grid size becomes finer. We also compared performance of the algorithms using Becker’s models 1 and 2 in Table 2 with *q* = 3. For Becker’s model 2, we used the same setup for comparing the Cocktail algorithm and the YBT algorithm in [1], which is what in the last example. With a grid of size 1001 for each factor, the YBT algorithm produced a design with 11 points at

$$\begin{array}{ccccccccccc} {\tilde{x}}_{1} & {\tilde{x}}_{2} & {\tilde{x}}_{3} & {\tilde{x}}_{4} & {\tilde{x}}_{5} & {\tilde{x}}_{6} & {\tilde{x}}_{7} & {\tilde{x}}_{8} & {\tilde{x}}_{9} & {\tilde{x}}_{10} & {\tilde{x}}_{11}\\ 0.0000 & 0.0010 & 0.0000 & 0.0000 & 0.0010 & 0.3330 & 0.3340 & 0.5000 & 0.5000 & 0.9990 & 0.9990\\ 0.0010 & 0.0000 & 0.5000 & 0.9990 & 0.9990 & 0.3330 & 0.3330 & 0.0000 & 0.5000 & 0.0000 & 0.0010\\ 0.9990 & 0.9990 & 0.5000 & 0.0010 & 0.0000 & 0.3340 & 0.3330 & 0.5000 & 0.0000 & 0.0010 & 0.0000 \end{array}$$

and the weight vector is (0.0714, 0.0714, 0.1429, 0.0714, 0.0714, 0.0714, 0.0714, 0.1429, 0.1429, 0.0714, 0.1429). This design is close to the *D*-optimal design found by ProjPSO shown in Table 2 and has a relative *D*-efficiency of 0.9974, implying that the ProjPSO-generated design is more efficient. The design found by the Cocktail algorithm is similarly highly efficient. The impact of the grid size on the optimal designs found from the Cocktail and YBT algorithms are shown in Table 4. For each of the 3 grid sizes, the table reports the number of design points found by each algorithm, the *D*-efficiency of the generated design relative to the ProjPSO-generated design and the CPU time. Results show a general trend that a finer grid size always produces designs with higher *D*-efficiencies. For this problem, the ProjPSO-generated 7-point design was found using an arbitrary number of 1024 particles and an arbitrary number of 400 iterations. It took 248.793 seconds to run, but the same design can also be found by ProjPSO with 200 or fewer iterations.

**Table 4:** Number of support points in the generated designs from the Cocktail and Optimal Weights Exchange algorithms using different grid sizes, their relative *D*-efficiencies and CPU times for the Becker’s Model 2.

Grid size	101	501	1001	2001
CA	12 points, 0.9747, 0.734 secs	12 points, 0.9949, 19.312 secs	12 points, 0.9974, 83.062 secs	10points, 0.9987, 357.750 secs
Yang	12 points, 0.9747, 2.665 secs	12 points, 0.9949, 36.074 secs	11 points, 0.9974, 207.143 secs	10points, 0.9987, 700.814 secs

We note that the grid points (1, 0, 0), (0, 1, 0) and (0, 0, 1) are not feasible for the Cocktail and YBT algorithms and so they can only identify the grid points close to these corner point. In contrast, the ProjPSO algorithm assumed a continuous design space and was able to identify design points very close to the 3 corner points. More specifically, ProjPSO was able to determine, for example, (1.0000, 1.91e-019, 0), (0, 1.0000, 3.48e-017) and (0, 4.56e-018, 1.0000) as design points. As a concrete example, consider the Becker’s Model 1. Table 5 shows designs generated by the Cocktail and YBT algorithms using different grid sizes are all highly *D*-efficient relative to the *D*-optimal design found by ProjPSO. The YBT algorithm required shorter CPU times when we have larger grid sizes. The CPU time required for ProjPSO to find the *D*-optimal design using 1024 particles and 400 iterations is around 243.633 seconds.

**Table 5:** Number of support points in the generated designs from the Cocktail and Optimal Weights Exchange algorithms using different grid sizes, their relative *D*-efficiencies and CPU times for the Becker’s Model 1.

Grid size	101	501	1001
CA	9 points, 1.0000, 0.937 secs	9 points, 1.0000, 87.281 secs	9 points, 1.0000, 1153.880 secs
Yang	9 points, 1.0000, 5.374 secs	9 points, 1.0000, 103.417 secs	9 points, 1.0000, 395.564 secs

**Multistage design:** [1] demonstrated that the YBT algorithm can also be applied to search for a multistage design. Suppose that we have an initial exact design *ξ*_0_ with *n*_0_ observations and based on its results, we want to augment the design by another *ξ* with *n*_1_ more observations so that the combined design *ξ*_0_ + *ξ* is optimal for a pre-specified design criterion. To show ProjPSO can also find such a multistage design, we only need to incorporate information from *ξ*_0_ when we calculate the information matrix in the code. Specifically, we choose *ξ*_1_ so that as a function of the weighted combination of the information matrices from *ξ*_0_ and the second stage design *ξ*_1_, the *D*-optimality criterion is optimized.

We demonstrate this procedure using Becker’s model 1 with *q* = 3. We first show the design obtained by the YBT algorithm and compare it with the one from ProjPSO after appropriate modification. Suppose the initial design, *ξ*_0_, is equally supported at 8 points

$$\begin{array}{cccccccc} x_{i,1} & x_{i,2} & x_{i,3} & x_{i,4} & x_{i,5} & x_{i,6} & x_{i,7} & x_{i,8}\\ 0.1 & 0.2 & 0.3 & 0.4 & 0.5 & 0.6 & 0.7 & 0.8\\ 0.8 & 0.5 & 0.2 & 0.5 & 0.1 & 0.2 & 0.3 & 0.1\\ 0.1 & 0.3 & 0.5 & 0.1 & 0.4 & 0.2 & 0.0 & 0.1 \end{array}$$

To improve this initial design (that’s what the subscript *i* is for in the above notation for the support points), we add *n*_1_ = 8 more observations so that the combined design is *D*-optimal. Following [1], we discretized the simplex design space into 1001 uniformly spaced points for each factor, ran the YBT algorithm and obtained the design with 8 points at

$$\begin{array}{cccccccc} {\tilde{x}}_{1} & {\tilde{x}}_{2} & {\tilde{x}}_{3} & {\tilde{x}}_{4} & {\tilde{x}}_{5} & {\tilde{x}}_{6} & {\tilde{x}}_{7} & {\tilde{x}}_{8}\\ 0.0000 & 0.0000 & 0.0000 & 0.0000 & 0.4380 & 0.4870 & 0.4880 & 1.0000\\ 0.0000 & 0.4860 & 0.4870 & 1.0000 & 0.5620 & 0.0000 & 0.0000 & 0.0000\\ 1.0000 & 0.5140 & 0.5130 & 0.0000 & 0.0000 & 0.5130 & 0.5120 & 0.0000 \end{array}$$

with the weight vector (0.1992, 0.1463, 0.0417, 0.1768, 0.0830, 0.1382, 0.0386, 0.1762). Clearly, there are two support points, namely ${\tilde{\text{x}}}_{6}$ and ${\tilde{\text{x}}}_{7}$, which can and should be merged. Using 1024 particles and 400 iterations, the ProjPSO algorithm found *ξ*_1_ to have the following support points:

$$\begin{array}{cccccc} {\tilde{x}}_{1} & {\tilde{x}}_{2} & {\tilde{x}}_{3} & {\tilde{x}}_{4} & {\tilde{x}}_{5} & {\tilde{x}}_{6}\\ 0.0000 & 0.4874 & 0.0000 & 0.0000 & 1.0000 & 0.4385\\ 0.0000 & 0.0000 & 1.0000 & 0.4864 & 0.0000 & 0.5165\\ 1.0000 & 0.5126 & 0.0000 & 0.5136 & 0.0000 & 0.0000 \end{array}$$

and the weight vector (0.1762, 0.1768, 0.1769, 0.1880, 0.1992, 0.083). In summary, the best second stage design given the exact design *ξ*_0_ is to augment it by *ξ*_1_ so that the new design is *D*-optimal for estimating the 7 parameters in Becker’s Model 1.

## Discussion

There are discrepancies in the above results from different packages and algorithms. Some are minor and others are less minor. For instance, only the ProjPSO algorithm was able to determine the *D*-optimal design for Becker’s Model 2. One reason is that some algorithm or package does not use the equivalence theorem to confirm optimality of the generated design. For example, AlgDesign does not incorporate a stopping criterion based on the equivalence theorem. The SAS package is primarily interested in exact optimal designs and so does not have a theoretical way to check optimality. The stopping criterion in the Cocktail algorithm is based on an equivalence theorem but the generated design, while highly efficient, is frequently not optimal because the support points are restricted to be the pre-specified grid points used to discretize the design space. The same is true for other algorithms that require that the design space to be discretized.

Requiring that a grid set be pre-specified to search for the design points of the optimal design implies that the resulting generated design can only be supported at a subset of the grid points. A fine grid leads to a more accurate search for the optimal design but at the expense of computational time. For example, if there are only 3 factors in the mixture model, it takes only 2 seconds to generate a set of 2001 uniformly spaced points for each factor, but when there are 4 factors in the model, it takes around 19570 seconds to generate 201 equally spaced points for each factor in the simplex. ProjPSO works on the a continuous domain and differentiates itself from many other algorithms by not requiring the user to specify a candidate set of designs points before the search can begin. We view this feature of ProjPSO a distinct advantage over its competitors.

Other advantages of using a PSO-based algorithm for finding optimal designs for mixture models over current methods are (1) the time required to generate the optimal design is generally faster than current methods; (2) it can be used to find optimal designs for models not available in the common statistical software packages; for example, ProjPSO finds the *I*-optimal design for the cubic Scheffé model with 3 factors quickly and we were not able to find current packages that produce such an optimal design; (3) the method can be readily modified to directly find optimal designs for more complicated design problems, where the model is nonlinear or the design criterionis not differentiable; see, for example, [44] and [45], and (4) the basic PSO algorithm is widely available freely in MATLAB, C++ on several websites and they can be easily amended to find various optimal designs for different models. Our codes are also freely available to interested reader by writing to the second author.

PSO also compares favorably with other metaheuristic algorithms in one aspect. Many researchers from various fields frequently report that the tuning parameters in PSO seem easy to use and are not as sensitive as those in other metaheuristic algorithms. For example, in genetic algorithms (GA) and simulated annealing (SA), all tuning parameters have to be carefully selected before the algorithms work well, see for example http://www.swarmintelligence.org/tutorials.php. Our experience is similar. Following convention, we use the default values in ProjPSO and set *γ*_1_ = *γ*_2_ = 2 to successfully search for the various optimal designs for our mixture design problems. The only exception was the case when we wanted to find a *D*-optimal design for the log contrast models where setting *γ*_1_ = *γ*_2_ = 0.5 seems to work better than the default values with *γ*_1_ = *γ*_2_ = 2.

Our experience with ProjPSO suggests that for finding an optimal approximate design, only two parameters in the ProjPSO algorithm seem to matter; the flock size and the number of iterations. The rest of the parameters can be set to their default values. A larger size of randomly generated flock of birds covers a broader range of the search space and so is suitable for more complex and high dimensional problems. A larger number of iterations minimizes the chance of early termination and allows ProjPSO additional time to find the optimum, which it usually does not need for solving our design problems. Our typical value for a flock size is 256 or 512 for more complex models and smaller for less complex models. A typical maximum iteration number that we used is 300.

We plan to do follow up work in [46] and [47] and modify the ProjPSO to search for multiple-objective optimal designs for mixture models and optimal designs for mixture amount models. Multiple-objective optimal designs are desirable because they can incorporate multiple goals of the study at the design stage and deliver a design with efficiencies specified by the user, with more important goals having larger efficiencies. Mixture amount mixture problems are useful because the optimal allocation schemes also depend on the total amount of resources available for the experiment [48]. We hope to report our results soon.
